# Diagnostic Accuracy of the Triglyceride–Glucose Index (TyG), TyG Body Mass Index, and TyG Waist Circumference Index for Liver Steatosis Detection

**DOI:** 10.3390/diagnostics14070762

**Published:** 2024-04-03

**Authors:** Alejandra Mijangos-Trejo, Raúl Gómez-Mendoza, Martha Helena Ramos-Ostos, Graciela Castro-Narro, Misael Uribe, Eva Juárez-Hernández, Iván López-Méndez

**Affiliations:** 1 Gastroenterology and Obesity Unit, Medica Sur Clinic & Foundation, Mexico City 14050, Mexico; almamitre@hotmail.com (A.M.-T.); raul.93gome@gmail.com (R.G.-M.); muribe@medicasur.org.mx (M.U.); 2 Diagnosis and Treatment Unit, Medica Sur Clinic & Foundation, Mexico City 14050, Mexico; mtramos@medicasur.org.mx; 3 Transplants and Hepatology Unit, Medica Sur Clinic & Foundation, Mexico City 14050, Mexico; gracastron@yahoo.com; 4 Translational Research Unit, Medica Sur Clinic & Foundation, Mexico City 14050, Mexico

**Keywords:** triglycerides, body mass index, steatosis, liver, waist circumference

## Abstract

Background: The triglyceride–glucose index (TyG) and a combination of body mass index (BMI) and waist circumference (WC) have been proposed as predictive scores for liver steatosis (LS). The aim of this study was to determine the diagnostic accuracy of these indices compared with controlled attenuation parameters (CAPs) and other predictive scores of LS. Methods: A retrospective analysis of patients who attended a check-up unit in 2021 was performed. LS was determined by CAP. Anthropometric and biochemical parameters for calculating TyG, TyG-BMI, TyG-WC, fatty liver index, and hepatic steatosis index were obtained. ROC curve was used to establish the best cut-off point of each TyG index for LS detection. The accuracy was determined for all patients, as well as for overweight and diabetic patients. Results: Medical records of 855 patients with a median age of 48 [IQR, 44–54] years and a BMI of 25.7 [IQR 23.4–28.1] kg/m^2^ were included. According to CAP, LS prevalence was 31.8% (*n* = 272). TyG-BMI and TyG-WC show better AUCs compared with CAP (0.82, 0.81), FLI (0.96, both), and HSI (0.93, 0.85). For diabetic patients, TyG-WC shows an AUC of 0.70. Meanwhile, TyG-BMI shows better accuracy (0.75) compared with CAP. Conclusions: TyG-BMI and TyG-WC showed a superior predictive accuracy for detecting LS compared with the TyG index.

## 1. Introduction

Liver steatosis is a common entity in chronic liver diseases. One of the most important and a public health issue is Metabolic-Associated Steatotic Liver Disease (MASLD), in which evidence of liver steatosis is the first step for diagnosis, followed by evidence of cardiometabolic abnormalities such as triglycerides and fasting glucose impairments [1]. MASLD is considered part of the metabolic syndrome spectrum with a closer relationship with insulin resistance [2,3] and with an important correlation with extrahepatic diseases such as diabetes mellitus (DM) and cardiovascular diseases [4]. As part of the natural history of MASLD, progression to fibrosis and cirrhosis has positioned MASLD as one of the most important causes of liver cirrhosis and hepatocellular carcinoma [5,6]. Nowadays, MASLD is the major cause of chronic liver disease worldwide, with a prevalence of 25.4% with variable ranges according to the geographical region [7].

The reference standard for liver steatosis diagnosis is liver biopsy; however, it is an invasive and expensive method, with a risk of sampling bias and interobserver variability. Due to the above and because performing a biopsy is not free of complications, some non-invasive tests have been developed and validated [8]. Among the imaging studies for detecting liver steatosis is ultrasound, which offers a sensitivity of 67–94% and a specificity of up to 97% [9]. Computed tomography has a sensitivity of 82% and a specificity of 100% to detect liver steatosis when the fat content is greater than or equal to 30%. However, tomography is not very useful for detecting mild levels of steatosis, and the radiation that the patient receives must be considered [10]. Magnetic resonance imaging (MRI) is the most sensitive imaging method to detect increased liver fat; this method has an accuracy close to 100% for detecting steatosis. There is a good correlation between MRI and histology in patients with liver steatosis, and steatosis can be detected with only 3% fat content. Among the disadvantages of resonance are the high cost and low availability of spectroscopy [10]. Controlled attenuation parameter (CAP) delivered by transient elastography (TE) is a non-invasive method with higher availability that has shown similar diagnostic accuracy to MRI, with a sensitivity of 87% for detecting liver steatosis [11].

The liver is the main organ responsible for glucose and lipid metabolism; it has been seen that patients with liver steatosis have elevated levels of free fatty acids (FFAs) due to a failure to suppress lipolysis mediated by insulin, allowing the release of excess FFAs into the bloodstream [2,12]. The increased flow of FFAs stimulates the secretion of very-low-density lipoprotein (VLDL), which prevents the adequate elimination of triacylglycerol, causing the development of hypertriglyceridemia and insulin receptor dysfunction [12,13]. Due to these biochemical mechanisms, in addition to the previously mentioned methods, specific scores that combine different clinical and biochemical variables for detecting liver steatosis and the risk of MASLD have been studied. Fatty liver index (FLI) incorporates the patient’s body mass index (BMI), waist circumference (WC), gamma-glutamyl transferase (GGT) levels, and serum triglyceride levels. The cut-off point for detecting liver steatosis with FLI is a value ≥60, with an AUC of 0.84 (95%CI 0.81–0.87), with a sensitivity of 61% [14]. On the other hand, the hepatic steatosis index (HSI) combines body mass index (BMI), the presence of type 2 diabetes mellitus (DM), gender, and transaminase levels; a value ≥ 36 indicates liver steatosis with an AUC of 0.81 (95%CI 0.80–0.82) and a sensitivity of 93.1% [9,15,16].

The triglyceride–glucose (TyG) index has recently been proposed as a simple and low-cost surrogate marker of insulin resistance (IR), and it has also recently been associated as a marker of MASLD, with an AUC of 0.90 with a cut-off point of 8.3, with a sensitivity of 72.2% [16,17,18,19]. This index may be especially useful in liver steatosis due to the importance of increased serum levels of triglycerides in the pathophysiology of lipid accumulation in liver tissue [17,20]. The index is calculated according to Ln [Tg (mg/dL) × fasting glucose (mg/dL)/2] [19]. Likewise, other combinations of the TyG index have been evaluated together with BMI (TyG-BMI), which is calculated by multiplying TyG index × BMI, and TyG index combined with waist circumference (TyG-WC) [(TyG index) × (waist circumference (cm)]. According to AUC, the diagnostic accuracy of these combined indices has been estimated as 0.80 for TyG-BMI with a cut-off of 237.7 and 0.81 with a sensitivity of 79.3% and for TyG-WC with a cut-off of 822.3, with a sensitivity of 80.8% [20,21,22,23]. The usefulness of these scores has been evaluated in obese and DM patients [21]. However, cut-off points are not well established due to the heterogeneity and characteristics of the studied population (majorly Asian).

Evaluating the diagnostic accuracy of these scores compared with other non-invasive methods is necessary to consider them as additional, easy, simple, more accessible, and low-cost options that allow the detection of liver steatosis in patients. Therefore, the aim of this study is to determine the diagnostic accuracy of the TyG, TyG-BMI, and TyG-WC indices for the detection of liver steatosis compared with CAP, as well as to determine their diagnostic accuracy compared with other predictive scores (FLI and HSI).

## 2. Materials and Methods

### 2.1. Study Population

In this medical record retrospective analysis, we included patients ≥18 years old who attended the check-up unit at Medica Sur Clinic & Foundation between January and December 2021; the exclusion criteria were a history of another chronic liver disease (viral, genetic, autoimmune, and drug-induced liver disease), human immunodeficiency virus, history of hepatotoxic treatments, and significant alcohol consumption (>2 drinks per day in women and >3 drinks per day in men). The absence of any viral, genetic, autoimmune, or drug-induced liver disease was confirmed by laboratory tests and medical history during the check-up. In addition, patients with studies that did not meet the TE quality criteria (IQR dB > 40, IQR kPa > 30, and correct probe according to BMI (>27 kg/m^2^ for XL probe)) as well as the presence of clinical significative fibrosis (>8 kPa) were also excluded.

Anthropometric parameters of waist circumference, weight, and height were collected, and BMI was calculated as weight (kg)/(height (m)^2^; the presence of overweight was determined as BMI ≥ 25 kg/m^2^. Fasting metabolic biochemical parameters (glucose, HbA1c, triglycerides, total cholesterol, high-density lipoprotein, aspartate aminotransferase (AST), alanine aminotransferase (ALT), and GGT) were collected.

### 2.2. Liver Steatosis Assessment

All patients underwent TE (Fibroscan 502 Touch, Echosens, Paris, France) to determine the presence of liver steatosis and fibrosis. TE was performed by a single experienced operator and was conducted according to the manufacturer’s recommendations. The presence of liver steatosis was determined with CAP, and the cut-off point of 268.5 dB/m was used, according to what was proposed by Cao et al. [24].

Additionally, five different scores for liver steatosis detection were determined in all patients according to established formulas:

(1) FLI: (FLI = (e^0.953*loge(triglycerides)+0.139*BMI+0.718*loge (GGT)+0.053*WC−15.745^)/(1 + e^0.953*loge(triglycerides)+0.139*BMI+0.718*loge (GGT)+0.053*WC−15.745)^ × 100) [14];
(2) HIS: (HSI = 8 × ALT/AST + BMI(+2 if type 2 DM present, +2 if female)) [15];
(3) TyG: Ln [Tg (mg/dL) × fasting glucose (mg/dL)/2] [19];
(4) TyG-BMI: (TyG index × BMI) [25];
(5) TyG-WC (TyG index) × (waist circumference (cm)) [25].

### 2.3. Statistical Analysis

Normality testing was conducted (Kolmogorov–Smirnov test), and distribution resulted as non-parametric; therefore, continuous variables were described using median and interquartile ranges, while categorical data were presented as numbers and percentages. Since the cut-off points of TyG, TyG-BMI, and TyG-WC are not well established, we carried out an analysis to determine the best cut-off point of these according to AUROC and Youden index, with respect to liver steatosis determination by CAP. With these values, the diagnostic accuracy of the ability of TyG, TyG-BMI, and TyG-WC to detect liver steatosis in patients was determined according to sensitivity, specificity, positive and negative predictive values, and positive and negative likelihood ratios. Accuracy was determined using CAP, FLI, and HSI as references. Once AUCs were determined, these were compared using the De Long method [26]. First, the diagnostic accuracy was determined in all patients. Then, we performed a sub-analysis to determine the diagnostic accuracy of the three indices for DM and overweight/obese patients (BMI ≥ 25 kg/m^2^).

A statistical analysis was performed using IBM SPSS Statistics for Mac, version 26 (IBM Corp., Armonk, NY, USA) software, and MedCalc Statistical Software, version 19.2.6 (MedCalcSoftware Ltd., Ostend, Belgium; https://www.medcalc.org; 2020), for ROC curve comparison.

## 3. Results

### 3.1. Study Population

Medical records of 2273 patients were collected. After applying the selection criteria, 855 patients were included in the analysis. In total, 61% (*n* = 522) of the patients were men with a median age of 48 years [IQR, 44–54] and a median BMI of 25.7 [IQR 23.4–28.1] kg/m^2^. The prevalence of overweight/obese patients was 59% (*n* = 505). The median of dB/m was 243 [IQR 213–282]. The prevalence of liver steatosis was 31.8% (*n* = 272), according to CAP, with FLI in 27.4% (*n* = 234) and HSI in 82.8% (*n* = 708). The median of liver stiffness was 3.9 (IQR, 3.3–4.6). The prevalence of DM was 6.3% (*n* = 54). The mean of TyG, TyG-BMI, and TyG-WC indices were 4.6 (4.4–4.8), 118.5 (106.1–133.5), and 425.9 (384.6–467.8), respectively. Table 1 presents the clinical characteristics of the population studied.

### 3.2. Diagnostic Accuracy of TyG Index, TyG-BMI Index, and TyG-WC Index

**Figure 1:**
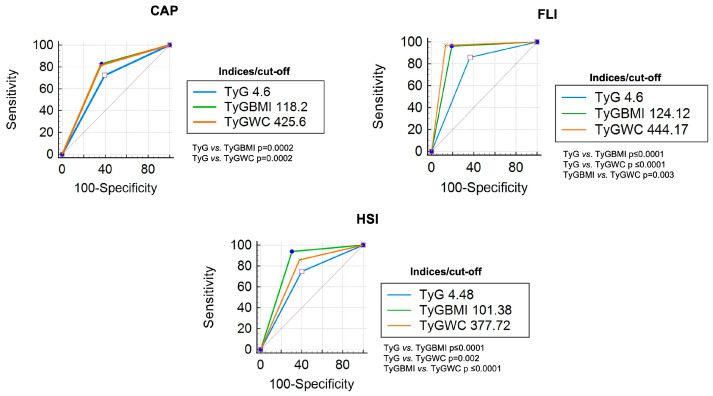
ROC curve comparison (all patients) of each TyG index with CAP, FLI, and HSI as references.

Compared with the CAP measurement, the TyG index had a sensitivity of 72% and a specificity of 60%, with an AUC of 0.72 for detecting liver steatosis, using a cut-off point of ≥4.61. Regarding the TyG-BMI index, the AUC was 0.82 with a cut-off point of ≥118.2. TyG-WC index had an AUC of 0.81 with a cut-off point of ≥425.6. In the ROC curve comparison, the TyG vs. TyG-BMI and TyG-WC show significant differences (*p* < 0.05); the comparison between TyG-BMI and TyG-WC did not show significant differences (Figure 1). Post-test probabilities of each index were 46% for TyG, 51% for TyG-BMI, and 52% for TyG-WC.

When the indices were compared with FLI, a cut-off point of ≥4.6 of the TyG index had a sensitivity of 85%, specificity of 63%, and AUC of 0.84 for the detection of liver steatosis; both the TyG-BMI and TyG-WC indices obtained AUCs of 0.96. All comparisons in the ROC curve showed significant differences (*p* < 0.05) (Figure 1). The post-test probability was higher for TyG-BMI (65%) and TyG-WC (73%) than TyG (47%).

With respect to HSI, an AUC of 0.74 was obtained with a cut-off point of ≥4.4 for the TyG index; the accuracy of TyG-BMI was 0.93 with a cut-off point of ≥101.38. Meanwhile, the sensitivity of TyG-WC was 91%, with a cut-off point of ≥377.7. All ROC curve comparisons show significant differences (*p* < 0.05) (Figure 1); the post-test probabilities were higher than 90% in all indices.

Table 2 shows the AUC, sensitivity, specificity, positive and negative predictive values, and likelihood ratios of each index when compared with CAP, FLI, and HSI. 

### 3.3. Diagnostic Accuracy of TyG Index, TyG-BMI Index, and TyG-WC Index in Patients with DM

An analysis of the diagnostic accuracy of each index was performed in patients with DM (*n* = 54). Characteristics of these patients are presented in Table 1. Compared with CAP, the TyG index obtained an AUC of 0.63, the TyG-BMI index obtained 0.68, and the TyG-WC index obtained 0.70. Post-test probabilities were higher in TyG (66%) and TyG-WC (68%) than in TyG-BMI (56%). Both the TyG-BMI and TyG-WC indices obtained an AUC greater than 0.95 when compared with FLI and HSI, with post-test probabilities higher than 70% when compared with FLI and higher than 90% compared with HSI. The TyG-BMI cutoff of ≥124.6 had an AUC of 0.97 compared with FLI, and a cutoff of ≥98.3 had an AUC of 0.99 compared with HSI. On the other hand, the TyG-WC index had an AUC of 0.96 with a cut-off point of 451.3 when compared with FLI and 0.95 with a cut-off point of 372.6 when compared with HSI (Table 3).

**Figure 2:**
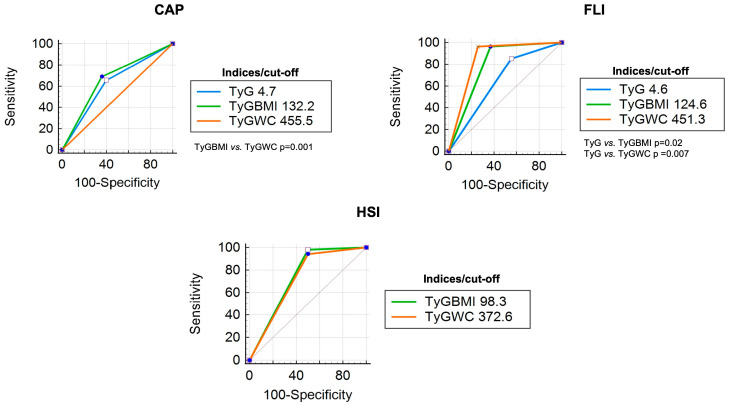
ROC curve comparison (DM patients) of each TyG index with CAP, FLI, and HSI as references.

According to the ROC curve comparison, when indices were compared with CAP as a reference, only ROC curves of TyG-BMI and TyG-WC showed a difference (*p* = 0.01); when FLI was used as a reference, significant differences were observed between TyG vs. TyG-BMI (*p* = 0.02) and TyG vs. TyG-WC (*p* = 0.007). In the curve comparison with HSI as the reference, all comparisons showed significant differences (*p* < 0.05) (Figure 2).

### 3.4. Diagnostic Accuracy of TyG Index, TyG-BMI Index, and TyG-WC Index in Overweight/Obese Patients

**Figure 3:**
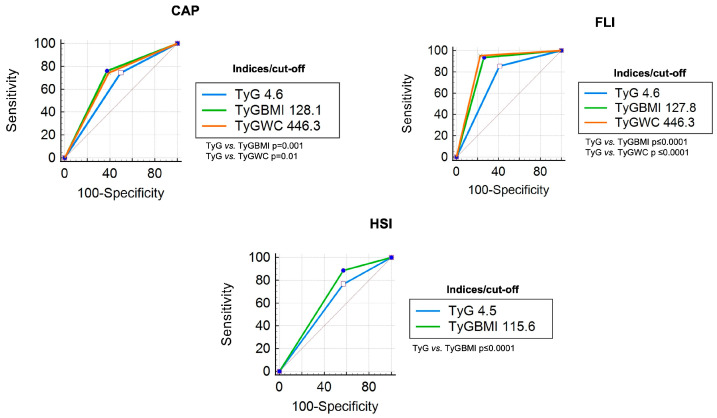
ROC curve comparison (overweight/obese patients) of each TyG index with CAP, FLI, and HSI as references.

A total of 505 patients presented a BMI ≥ 25 kg/m^2^. Characteristics of these patients are presented in Table 1. Compared with CAP, the TyG index obtained an AUC of 0.67, the TyG-BMI index obtained 0.75, and the TyG-WC index obtained 0.74; differences between ROC curves were observed in TyG vs. both TyG-BMI and TyG-WC, *p* = 0.001 and *p* = 0.01, respectively (Figure 3).

In contrast, compared with FLI, the TyG-BMI index had an AUC of 0.92 with a cut-off point of 127.8, and the TyG-WC index had an AUC of 0.95 with a cut-off point of 446.3, The AUC of TyG (cut-off 4.6) was 0.85 with a sensitivity of 85%; TyG-BMI vs. TyG-WC did not show significant differences in ROC curve comparative analysis (Figure 3). When HSI was used as a reference, the TyG-WC ROC curve crossed the reference line. Therefore, only TyG and TyG-BMI were analyzed with a sensitivity of 76% and 88%, respectively, with a significant difference in ROC curve comparison (*p* ≤ 0.0001) (Figure 3). In this analysis, post-test probabilities were higher for TyG-BMI and TyG-WC indices (64–99%). The diagnostic accuracy of each index in overweight and obese patients is shown in Table 4. 

## 4. Discussion

Liver steatosis has become the cornerstone of the most common chronic liver disorders and has been found to have a close association with metabolic syndrome components [8]. Serum triglyceride and glucose levels may be especially useful in the diagnosis of liver steatosis due to the importance of insulin resistance and increased serum levels of triglycerides in the pathophysiology of lipid accumulation in the liver tissue [2,17].

In this study, different parameters based on serum triglyceride and glucose levels were tested to determine the presence of liver steatosis. Considering the role of overweight and obesity in the development of IR and MASLD, it was hypothesized that by adding anthropometric markers (such as BMI and WC) to the TyG index, better indicators of liver steatosis would be obtained, which could be proven by our findings. When compared with CAP, we observed that the TyG index had the lowest AUC (0.72) for detecting liver steatosis, and the best AUC was observed with the TyG-BMI index (AUC 0.82). Importantly, in most comparisons, the best diagnostic accuracy was obtained with the TyG-BMI index, followed by the TyG-WC index. The TyG index obtained lower accuracy than the other indices evaluated, so we could highlight that adding anthropometric parameters to the TyG index increases diagnostic accuracy. These findings were obtained with cut-off points different from those previously reported; we performed an analysis to determine the best cut-off point for our population since most of the evidence about the usefulness of these indices comes from Asian populations, which have different anthropometric characteristics and risk factors than the occidental population.

Several studies have reported the usefulness of the TyG index for liver steatosis screening in large, healthy populations [17,27,28]. However, most of these studies were conducted in an Asian population.

In 2014, Fedchuk et al. [16] published one of the first studies on the TyG index and the presence of MASLD (formerly named non-alcoholic fatty liver disease (NAFLD)). They reported an AUC of 0.90, sensitivity of 80%, and specificity of 69%, using a cut-off point of 8.38, compared with liver biopsy. However, 41% of the population had DM, and 24% had advanced fibrosis in that study. In our study, the prevalence of DM was 6.3%, and patients with advanced fibrosis were excluded. The differences in population characteristics can be attributed to the fact that the study published by Fedchuk et al. included patients who underwent a biopsy for suspected NAFLD, and our study population included patients who attended a health check-up in whom there was no prior suspicion of liver steatosis.

A meta-analysis carried out by Jing Wang et al. [20] observed that the TyG index has a sensitivity of 0.73 (95% CI 0.69–0.76), specificity of 0.67 (95% CI 0.65–0.70), and an AUC of 0.75 (95% CI 0.71–0.79) for liver steatosis detection. The sensitivity, specificity, and AUC reported by Wang et al. were higher than our findings. However, we must consider that Jing Wang’s meta-analysis included studies where the comparison of diagnostic accuracy was with ultrasound. An additional consideration is that ultrasound has the limitation of being an operator-dependent study, unlike CAP.

Concerning TyG-BMI index, we were able to demonstrate greater accuracy in the diagnostic prediction of liver steatosis compared with the TyG index, which agrees with what was reported by a study carried out by Mengyuan Wang et al. [29], where they also observed that the TyG-BMI index was more closely related to liver steatosis severity. In 2023, a Chinese study also reported a greater predictive capacity of the TyG-BMI index for detecting liver steatosis than TyG and TyG-WC indices, especially in the lean and female populations [28].

Li et al. provided evidence that the TyG-BMI index outperforms the TyG index in predicting the presence of NAFLD in patients with DM, which is consistent with the results we obtained [30]. Furthermore, this information is supported by the group of Lim et al. [31], who demonstrated that the TyG-BMI index was also superior in predicting IR compared with the TyG index and the TyG-WC index.

The TyG-WC index is a new IR indicator that reflects both visceral fat and liver steatosis. Kim et al. [22] demonstrated that the TyG-WC index was superior to the HOMA-IR in detecting NAFLD in healthy Korean adults, especially in the non-obese population. Therefore, subsequent studies were carried out where it was demonstrated that TyG-WC is a reliable indicator of the presence of NAFLD [32,33,34]. However, according to what was observed in our study population, the TyG-WC index could also be used in obese and overweight patients to predict the presence of liver steatosis. An Iranian study supports this information since it demonstrated that the TyG-WC index was one of the best indicators of the presence of NAFLD in overweight/obese people without DM, and it was also reported that it could indicate the presence of liver fibrosis [25].

One of the main strengths of our study was that, in addition to evaluating the diagnostic accuracy of the TyG index, the TyG-BMI and TyG-WC indices were also assessed. Furthermore, most previous studies only compared the diagnostic accuracy with ultrasound, CAP, or FLI. In our study, the diagnostic accuracy was compared with CAP, FLI, and HSI.

However, this study has several limitations. First, this is a single-center study conducted in the Mexican population, so the information cannot be generalized to other populations. Future studies should include heterogeneous populations to ensure generalization to other ethnicities. Second, this study had a retrospective cross-sectional design, with the respective limitations that this entails. On the other hand, the population for this study was selected in a check-up unit and this could represent a selection bias for the extrapolation of results. It should also be considered that liver steatosis was not measured by biopsy, which is considered the reference standard for diagnosis. However, CAP has a high concordance with liver biopsy for diagnosing and classifying liver steatosis, which is why it has been endorsed by leading guidelines as the preferred non-invasive diagnostic instrument for evaluating liver steatosis [35,36].

According to our results and in concordance with previous reports, the TyG index has a lower accuracy for the detection of liver steatosis; meanwhile, TyG indices combined with anthropometric measurements (TyG-BMI and TyG-WC) show better accuracy for the detection of liver steatosis when these are compared with an non-invasive imaging method such as CAP, and this superior accuracy is maintained when these indices are compared with other predictor scores for liver steatosis (FLI and HSI). Therefore, in non-diabetic, general population screening, TyG-BMI and TyG-WC indices could be used indistinctly to detect liver steatosis.

With respect to DM and overweight/obese patients, TyG-BMI and TyG-WC indices also have better accuracy for the detection of liver steatosis. However, the performance of TyG-BMI is higher in these patients when HSI is used as a reference for diagnostic accuracy, with better AUC. This result could be explained since DM and BMI are part of the variables included in the HSI model, and both conditions are strongly associated with higher visceral fat and IR.

The highlight of these tools is that, with easily available biochemical and anthropometric determinations, they can be applied both in the general population and in patients considered at high risk (obese and DM). Their strength lies in their positive predictive value, that is, their inclusion of patients who, once identified, must be taken to a specific imaging method available according to each center’s needs and characteristics and continue the staging and treatment approach according to the current guidelines for MASLD.

## 5. Conclusions

Our results show that TyG indices combined with anthropometrical measurements (BMI and WC) are highly accurate in the detection of liver steatosis; thus, they are useful screening tools for identifying these patients, in whom a confirmatory imaging evaluation should be performed, and following the algorithm for MASLD diagnosis.

## Figures and Tables

**Table 1:** General characteristics of patients.

Characteristic	General (*n* = 855)	DM (*n* = 54)	Overweight/Obesity (*n* = 505)
	%(*n*)/M [IQR]	%(*n*)/M [IQR]	%(*n*)/M [IQR]
Male	61.1 (522)	63 (34)	69.1 (349)
Age (years)	48 (44–54)	54 (49–61)	49 (44–54)
BMI (kg/m^2^)	25.7 [23.4–28.1]	27.6 [25.3–30.9]	27.5 [26.1–29.7]
DM	6.3 (54)	-	8.9 (45)
Hypertension	35.1 (300)	48.1 (26)	44 (222)
Dyslipidemia	38.7 (331)	26.9 (16)	39.2 (198)
Smoking	20 (171)	16.7 (1)	21 (106)
WC (cm)	92 (85–98)	98 (91–106)	97 (92–103)
Fasting glucose (mg/dL)	90 (85–96)	111.5 (91–136)	91 (87–98)
Triglycerides (mg/dL)	112 (80–159)	116 [90.5–173.2]	126 [94.5–181.5]
Cholesterol (mg/dL)	208 (180–235)	194.5 (157–225)	208 (179–234)
HDL (mg/dL)	53 (42–74)	46.5 (41–81)	49 (39–68)
LDL (mg/dL)	131 (107–157)	115 [88.7–144.7]	131 (109–155)
HbA1c (%)	5.3 [5.1–5.5]	6.3 [5.5–7.3]	5.4 [5.2–5.7]
AST (IU/L)	26 (20–35)	25 (21–38)	28 (22–40)
ALT (IU/L)	23 (20–28)	24 (19–29)	24 (21–30)
GGT (IU/L)	21 (15–30)	21 (14–32)	24 (18–33)
dB	243 (213–282)	272 (243–309)	264 (238–302)
kPa	3.9 [3.3–4.6]	4.2 [3.6–4.7]	4.1 [3.4–4.6]
LS by CAP	31.8 (272)	53.7 (29)	46.3 (234)
FLI	37 (18–62)	59.5 [28.7–79.5]	55 (37–76)
LS by FLI	27.4 (234)	50 (27)	45.1 (228)
HSI	40 (43–47)	42.4 [39.8–46.8]	43 [40.5–46]
LS by HSI	82.8 (708)	96.3 (52)	98.6 (498)
TyG	4.6 [4.4–4.8]	4.7 [4.5–5.03]	4.6 [4.5–4.8]
TyG-BMI	118.5 [106.1–133.5]	133.8 [119.7–154.2]	129.9 [12.5–142.5]
TyG-WC	425.9 [384.6–467.8]	470.3 [427.4–508.7]	453.2 [424–495.3]

M [IQR], median [interquartile range]; DM, diabetes mellitus; BMI, body mass index; HDL, high-density lipoprotein; LDL, low-density lipoprotein; HbA1c, glycosylated hemoglobin; AST, aspartate aminotransferase; ALT, alanine aminotransferase; GGT, gamma glutamyl transpeptidase; dB, decibels; kPa, kilopascals; LS, liver steatosis; CAP, controlled attenuation parameter; FLI, fatty liver index; HSI, hepatic steatosis index; TyG, triglyceride–glucose index; TyG-BMI, triglyceride–glucose body mass index; TyG-WC, triglyceride–glucose waist circumference index.

**Table 2:** Diagnostic accuracy of TyG, TyG-BMI, and TyG-WC compared with CAP, FLI, and HSI in all patients (*n* = 855).

Index	Cut-Off	AUC	Sensitivity (CI 95%)	Specificity (CI 95%)	PPV	NPV	LR+ (CI 95%)	LR− (CI 95%)
CAP								
TyG	4.61	0.72	0.72 (0.67–0.77)	0.60 (0.54–0.66)	0.46	0.82	1.83 (1.61–2.07)	0.46 (0.37–0.56)
TyG-BMI	118.2	0.82	0.82 (0.77–0.87)	0.63 (0.57–0.69)	0.51	0.88	2.26 (2.01–2.55)	0.27 (0.21–0.36)
TyG-WC	425.6	0.81	0.81 (0.76–0.86)	0.64 (0.58–0.70)	0.51	0.88	2.29 (2.02–2.59)	0.29 (0.22–0.37)
FLI								
TyG	4.6	0.84	0.85 (0.80–0.90)	0.63 (0.57–0.69)	0.46	0.92	2.35 (2.09–2.64)	0.22 (0.16–0.31)
TyG-BMI	124.12	0.96	0.96 (0.93–0.99)	0.80 (0.75–0.85)	0.65	0.98	5.02 (4.26–5.91)	0.05 (0.03–0.09)
TyG-WC	444.17	0.96	0.97 (0.95–0.99)	0.86 (0.82–0.90)	0.73	0.98	7.26 (5.93–8.88)	0.03 (0.02–0.07)
HSI								
TyG	4.4	0.74	0.74 (0.71–0.77)	0.59 (0.55–0.63)	0.89	0.33	1.87 (1.52–2.28)	0.42 (0.35–0.50)
TyG-BMI	101.38	0.93	0.93 (0.91–0.95)	0.69 (0.66–0.72)	0.93	0.70	3.07 (2.40–3.92)	0.09 (0.06–0.12)
TyG-WC	377.7	0.85	0.85 (0.82–0.88)	0.61 (0.57–0.65)	0.91	0.47	2.25 (1.83–2.78)	0.23 (0.18–0.28)

PPV, positive predictive value; NPV, negative predictive value; LR, likelihood ratio; CAP, controlled attenuation parameter; TyG, triglyceride–glucose index; TyG-BMI, triglyceride–glucose body mass index; TyG-WC, triglyceride–glucose waist circumference index; FLI, fatty liver index; HSI, hepatic steatosis index.

**Table 3:** Diagnostic accuracy of TyG, TyG-BMI, and TyG-WC compared with CAP, FLI, and HSI in DM patients (*n* = 54).

Index	Cut-Off	AUC	Sensitivity (CI 95%)	Specificity (CI 95%)	PPV	NPV	LR+ (CI 95%)	LR− (CI 95%)
CAP								
TyG	4.7	0.634	0.65 (0.48–0.82)	0.60 (0.42–0.78)	0.34	0.40	1.64 (0.95–2.83)	0.57 (0.32–1.04)
TyG-BMI	132.2	0.683	0.69 (0.52–0.86)	0.36 (0.19–0.53)	0.44	0.50	1.08 (0.75–1.58)	0.86 (0.41–1.83)
TyG-WC	455.5	0.708	0.72 (0.56–0.88)	0.60 (0.42–0.78)	0.32	0.34	1.81 (1.07–3.08)	0.46 (0.24–0.90)
FLI								
TyG	4.6	0.771	0.85 (0.72–0.98)	0.44 (0.25–0.63)	0.39	0.25	1.53 (1.06–2.22)	0.33 (0.12–0.90)
TyG-BMI	124.6	0.975	0.96 (0.89–1.03)	0.63 (0.45–0.81)	0.27	0.05	2.60 (1.58–4.28)	0.06 (0.01–0.41)
TyG-WC	451.3	0.966	0.96 (0.89–1.03)	0.74 (0.57–0.91)	0.21	0.04	3.71 (1.95–7.96)	0.05 (0.01–0.35)
HSI								
TyG	-	-	-	-	-	-	-	-
TyG-BMI	98.3	0.990	0.98 (0.94–1.02)	0.50 (0.36–0.64)	0.01	0.05	1.96 (0.49–7.85)	0.04 (0.0–0.42)
TyG-WC	372.6	0.952	0.94 (0.88–1.00)	0.50 (0.36–0.64)	0.02	0.75	1.88 (0.47–7.55)	0.12 (0.02–0.68)

PPPV, positive predictive value; NPV, negative predictive value; LR, likelihood ratio; CAP, controlled attenuation parameter; TyG, triglyceride–glucose index; TyG-BMI, triglyceride–glucose body mass index; TyG-WC, triglyceride–glucose waist circumference index; FLI, fatty liver index; HSI, hepatic steatosis index.

**Table 4:** Diagnostic accuracy of TyG, TyG-BMI, and TyG-WC compared with CAP, FLI, and HSI in overweight and obese patients (*n* = 505).

Index	Cut-Off	AUC	Sensitivity (CI 95%)	Specificity (CI 95%)	PPV	NPV	LR+ (CI 95%)	LR− (CI 95%)
CAP								
TyG	4.6	0.674	0.74 (0.68–0.80)	0.50 (0.44–0.56)	0.43	0.30	1.49 (1.30–1.72)	0.51 (0.40–0.65)
TyG-BMI	128.1	0.756	0.76 (0.71–0.81)	0.62 (0.56–0.68)	0.36	0.24	2.02 (1.71–2.39)	0.38 (0.30–0.49)
TyG-WC	446.3	0.740	0.74 (0.68–0.80)	0.60 (0.54–0.66)	0.37	0.26	1.90 (1.61–2.25)	0.42 (0.33–0.53)
FLI								
TyG	4.6	0.827	0.85 (0.80–0.90)	0.58 (0.52–0.64)	0.36	0.16	2.08 (1.79–2.42)	0.25 (0.18–0.34)
TyG-BMI	127.8	0.929	0.93 (0.90–0.96)	0.73 (0.67–0.79)	0.25	0.06	3.54 (2.90–4.33)	0.09 (0.05–0.15)
TyG-WC	446.3	0.950	0.95 (0.92–0.98)	0.77 (0.72–0.82)	0.22	0.04	4.18 (3.36–5.21)	0.06 (0.03–0.11)
HSI								
TyG	4.5	0.695	0.76 (0.72–0.80)	0.42 (0.38–0.46)	0.01	0.97	1.34 (0.71–2.55)	0.54 (0.23–1.30)
TyG-BMI	115.6	0.884	0.88 (0.85–0.91)	0.57 (0.53–0.61)	0.006	0.93	2.07 (0.88–4.86)	0.20 (0.10–0.40)
TyG-WC	-	-	-	-	-	-	-	-

PPV, positive predictive value; NPV, negative predictive value; LR, likelihood ratio; CAP, controlled attenuation parameter; TyG, triglyceride–glucose index; TyG-BMI, triglyceride–glucose body mass index; TyG-WC, triglyceride–glucose waist circumference index; FLI, fatty liver index; HSI, hepatic steatosis index.

## Data Availability

The data presented in this study are available on request from the corresponding authors. The data are not publicly available due to privacy and ethics.
